# Resources Underlying Visuo-Spatial Working Memory Enable Veridical Large Numerosity Perception

**DOI:** 10.3389/fnhum.2021.751098

**Published:** 2021-11-15

**Authors:** Elisa Castaldi, Manuela Piazza, Evelyn Eger

**Affiliations:** ^1^Department of Neuroscience, Psychology, Pharmacology and Child Health, University of Florence, Florence, Italy; ^2^Department of Translational Research and New Technologies in Medicine and Surgery, University of Pisa, Pisa, Italy; ^3^Center for Mind/Brain Sciences, University of Trento, Rovereto, Italy; ^4^Cognitive Neuroimaging Unit, INSERM, CEA DRF/JOLIOT, Université Paris-Saclay, NeuroSpin Center, Gif-sur-Yvette, France

**Keywords:** numerosity perception, visuo-spatial working memory, approximate number system, saliency map, developmental dyscalculia, arithmetic

## Abstract

Humans can quickly approximate how many objects are in a visual image, but no clear consensus has been achieved on the cognitive resources underlying this ability. Previous work has lent support to the notion that mechanisms which explicitly represent the locations of multiple objects in the visual scene within a mental map are critical for both visuo-spatial working memory and enumeration (at least for relatively small numbers of items). Regarding the cognitive underpinnings of large numerosity perception, an issue currently subject to much controversy is why numerosity estimates are often non-veridical (i.e., susceptible to biases from non-numerical quantities). Such biases have been found to be particularly pronounced in individuals with developmental dyscalculia (DD), a learning disability affecting the acquisition of arithmetic skills. Motivated by findings showing that DD individuals are also often impaired in visuo-spatial working memory, we hypothesized that resources supporting this type of working memory, which allow for the simultaneous identification of multiple objects, might also be critical for precise and unbiased perception of larger numerosities. We therefore tested whether loading working memory of healthy adult participants during discrimination of large numerosities would lead to increased interference from non-numerical quantities. Participants performed a numerosity discrimination task on multi-item arrays in which numerical and non-numerical stimulus dimensions varied congruently or incongruently relative to each other, either in isolation or in the context of a concurrent visuo-spatial or verbal working memory task. During performance of the visuo-spatial, but not verbal, working memory task, precision in numerosity discrimination decreased, participants’ choices became strongly biased by item size, and the strength of this bias correlated with measures of arithmetical skills. Moreover, the interference between numerosity and working memory tasks was bidirectional, with number discrimination impacting visuo-spatial (but not verbal) performance. Overall, these results suggest that representing visual numerosity in a way that is unbiased by non-numerical quantities relies on processes which explicitly segregate/identify the locations of multiple objects that are shared with visuo-spatial (but not verbal) working memory. This shared resource may potentially be impaired in DD, explaining the observed co-occurrence of working memory and numerosity discrimination deficits in this clinical population.

## Introduction

Extracting estimates of the number of objects in a visual scene is important to guide many of our daily decisions. Much evidence suggests that numerosity perception is spontaneous and based on a non-verbal capacity which allows for judgments of the approximate number of objects at a glance, commonly termed “number sense” (Dehaene, 1997; Cantlon et al., 2009; Halberda et al., 2012; Anobile et al., 2016). Nevertheless, the precise perceptual and cognitive resources underlying this ability remain controversial.

For small numerosities within the subitizing range, i.e., up to four items, numerosity judgments are typically much more accurate compared to those for larger numbers (Kaufman et al., 1949; Mandler and Shebo, 1982; Revkin et al., 2008). However, the higher precision for small numerosities appears to depend on the availability of “domain general” cognitive resources such as working memory and attention (Burr et al., 2010; Melcher and Piazza, 2011; Piazza et al., 2011). One study found that the enumeration accuracy for small quantities in the subitizing range was affected by a concurrent visuo-spatial working memory task and that, vice versa, accuracy on the visuo-spatial task was lower when there were many compared to few items to enumerate (Piazza et al., 2011). This mutual interference, and the similar capacity limits measured across tasks, were interpreted to suggest that both visuo-spatial working memory and enumeration of small numbers of items may be supported by a basic mechanism of “visual indexing” of multiple objects, that is, a mechanism which allows us to simultaneously attend to multiple objects in parallel and explicitly represent their positions. This mechanism has been hypothesized to correspond to a mental map of the locations of salient objects in the visual scene, also referred to by the term “visual saliency map” (Koch and Ullman, 1987; Itti and Koch, 2000). In such a map, the saliency of individual objects is thought to be determined by either bottom-up (e.g., visual contrast) or top-down (e.g., task relevance) factors, and capacity limits of the map are flexible and determined by competitive interactions. In accordance with predictions from salience map theories, making one particular item more salient was found to reduce the subitizing range as well as memory performance for all other less salient items (Melcher and Piazza, 2011). The degree of involvement of such resources related to visuo-spatial working memory in the perception of larger numerosities remains unclear: although a small effect of performing a concurrent visuo-spatial working memory task was also found to decrease the precision of discrimination of larger numerosities (10–44 dots) by Piazza et al. (2011), the absence of a dependence of this effect on working memory load as well as the lack of another control task made it difficult to specifically attribute this effect to visuo-spatial working memory resources as opposed to more non-specifically enhanced cognitive load during dual task performance.

One issue which recently has given rise to much controversy on the cognitive underpinnings of “number sense” is the fact that performance in numerosity discrimination tasks can often non-veridically reflect the discrete number of items and instead be influenced by non-numerical properties of the sets (such as total luminance, area, density, and so on). When making non-numerical dimensions uninformative for the numerosity judgment, numerosity can still be discriminated, however, with lower accuracy when non-numerical dimensions vary incongruently with numerosity (e.g., Hurewitz et al., 2006; Nys and Content, 2012; Szûcs et al., 2013b; Salti et al., 2016). Moreover, non-numerical dimensions can bias behavioral choices in numerosity discrimination tasks, leading to consistent over or under estimation of numerosity. In adults these effects are typically subtle and mostly arise when the numerical ratios compared are rather small (Tokita and Ishiguchi, 2010; Nys and Content, 2012; DeWind et al., 2015) and become more evident when the variation in non-numerical dimensions is perceptually more salient than the numerical one (Hurewitz et al., 2006; Tokita and Ishiguchi, 2010; Gebuis and Reynvoet, 2011, 2012a,b). Recent studies have proposed that during development and/or arithmetical learning, children progressively learn to “focus on number” and to discard the influence of non-numerical quantities (Starr et al., 2017; Piazza et al., 2018). Interestingly, individuals with developmental dyscalculia (DD), a specific learning disability that prevents them from learning numerical and arithmetical skills (American Psychiatric Association, 2013), have not only been found to show decreased numerosity precision (Piazza et al., 2010; Mazzocco et al., 2011; Mejias et al., 2012; Anobile et al., 2018; Decarli et al., 2020) but be particularly impaired when non-numerical quantities provide incongruent information which tends to strongly bias their judgments (Szûcs et al., 2013a; Bugden and Ansari, 2016; Castaldi et al., 2018; Piazza et al., 2018). Some authors have attributed such findings to deficits in executive functions and more specifically, problems in inhibiting responses to task-irrelevant dimensions of the stimuli (Gilmore et al., 2013; Szûcs et al., 2013a; Bugden and Ansari, 2016). Nevertheless, our own findings in adults with DD showed that enhanced interference from unattended quantities was present only during numerosity comparisons, but not when subjects had to compare an orthogonal dimension (average item size) of the same stimuli (Castaldi et al., 2018), arguing against an impairment in general inhibitory skills as the source of the underlying problem.

More generally, it has been observed that difficulties in DD individuals span beyond the specific domain of numerical cognition: both DD children and adults often present working memory, attention and cognitive control deficits (Ashkenazi et al., 2013; Szûcs et al., 2013a; Menon, 2016; Castaldi et al., 2018, 2020b; Mammarella et al., 2018; Decarli et al., 2020, for reviews see: Fias et al., 2013; Iuculano, 2016; Castaldi et al., 2020a). With respect to working memory, a recent metanalysis showed that visuo-spatial working memory deficits characterize the “pure” DD subtype with respect to profiles with associated reading deficit (comorbid dyslexic dyscalculic disability), which are instead frequently associated with weak verbal working memory (Szûcs, 2016). Interestingly, Bugden and Ansari (2016) found that differences in numerosity precision and error rate during trials with incongruent non-numerical properties correlated with visuo-spatial working memory performance in DD children.

In the context of the reviewed findings on the observed co-existence of enhanced susceptibility to bias from non-numerical quantities and visuo-spatial working memory impairments in dyscalculia, together with the earlier mentioned evidence for potential shared resources between visuo-spatial working memory and enumeration at least of small numbers of items, we speculated that resources involved in visuo-spatial working memory might also be crucial for representing larger numerosities without bias from non-numerical quantities. Specifically, mechanisms as assumed by theories related to salience maps that explicitly represent the locations of multiple items and thereby allow for object segregation (as opposed to mechanisms that encode the visual scene in a mere gist-like, undifferentiated fashion) might be required to extract an unbiased representation of discrete numbers of items and such a representation might be relevant for arithmetical learning. While the co-occurrence of visuo-spatial working memory impairments and enhanced susceptibility to bias from non-numerical quantities in DD could still be explained by coexisting but functionally unrelated cognitive phenomena, we reasoned that to support a causal role of visuo-spatial working memory resources in veridical representation of large numerosities, manipulating the availability of working memory resources in neurotypical adults during performance of a numerosity task should interfere with numerosity judgments and lead to increasing perceptual biases from non-numerical stimulus dimensions.

In the current study, we therefore adapted the paradigm previously used to study numerosity perception and interference from non-numerical quantities in DD adults and controls by Castaldi et al. (2018), so that the numerosity discrimination task was performed either in isolation or in the context of a concurrent working memory task, and evaluated both numerosity precision and interference from the unattended size dimension (perceptual biases). Going beyond previous demonstrations of effects of working memory load on enumeration accuracy in the context of small sets of items, we further explicitly tested the specificity of the observed interference to the type of working memory resources: Given that visuo-spatial, but not verbal, working memory requires representing spatial locations of multiple items in parallel, we predicted that loading specifically visuo-spatial (but not verbal) working memory should give rise to imprecise and biased numerosity judgments. Moreover, if the systems supporting visuo-spatial working memory and numerosity perception share common resources, we expect a bidirectional interference between numerosity discrimination and working memory performance. Finally, if the shared resource contributing to visuo-spatial working memory and to veridical perception of numerosity is relevant also for more abstract arithmetical abilities, inter-individual differences in measures of arithmetical abilities should be predicted by the numerosity biases measured while participants’ visuo-spatial, but not verbal, working memory was loaded.

## Materials and Methods

Twelve adults with normal or corrected to normal vision (age 24 ± 3, 6 females) were included in the study. Prior to the study, written informed consent was obtained from all participants in accordance with the Declaration of Helsinki, and the study was approved by the research ethics committee of University Paris-Saclay. Prior to the study participants were asked whether they have ever encountered problems in learning math (or other school achievements, such as reading or writing), to qualitatively evaluate whether they might present learning disabilities. None of the participants reported having ever had such difficulties.

Participants were tested with seven conditions in which they performed different tasks: one baseline numerosity discrimination task, two single working memory tasks, two single and two dual tasks where numerosity discrimination was probed either in isolation or together with working memory, during presentation of identical stimuli (detailed below).

Participants sat in a dimly lit room at approximately 60 cm from a 15-inch Laptop (HP) with LCD monitor running at 60 Hz and with 1600 × 900 resolution. Visual stimuli were viewed binocularly and were generated under Matlab using PsychToolbox routines (Brainard, 1997).

### Baseline Numerosity Discrimination Task

The aim of this first condition was to measure participants’ numerosity discrimination performance at baseline, while being presented with no visual stimuli other than the ones for which a numerosity judgment was required. Stimuli were the same as the ones used in the numerosity task by Castaldi et al. (2018). Participants were presented with two heterogenous arrays of dots, half black and half white, displayed on a gray background so that luminance was not a cue for number. The arrays were simultaneously presented at the two sides of a central fixation point at 6 visual degrees (°) of eccentricity along the horizontal meridian. Individual dots were constrained to fall within a virtual circle of 5.8° or 7.6° diameter, to not overlap with the fixation point and to be at least 0.25° apart from each other. The test arrays contained 5, 6, 8, 12, 17, and 20 dots (ratios 0.5, 0.6, 0.8, 1.2, 1.7, 2 with respect to the reference of 10 dots). The arrays had either small (0.25°) or big (0.5°) average item diameter. Test stimuli were compared against a reference stimulus of 10 dots with 0.35° average item diameter with the same total field area as the test. Test and reference stimuli were presented either to the right or to the left of the central fixation point. The two arrays of dots were presented for 200 ms, followed by two questions. The first question asked the participant to report which of the two stimuli appeared more numerous (the question “number?” appeared onscreen). Participants were instructed to press either the left or the right arrow to provide the response. Then, a second question “same or different?” appeared onscreen, but in the current task participants were instructed to ignore it and to press the spacebar to move on to the next trial. Participants performed 12 practice trials, after which the experiment started. No feedback was provided during the practice trials, nor during the following experimental runs. Each participant performed three sessions. Each one of the 6 comparison ratios was presented 48 times: 2 average item size (small and big), 2 possible total field areas, 2 possible spatial positions with respect to the reference (left-right) repeated 2 times in each one of the 3 sessions. A total of 288 trials were collected and used for the analysis.

### Single Working Memory Tasks

Participants then performed two single working memory tasks that aimed at measuring the number of elements that could be held in both verbal and visuo-spatial working memory. The number of elements selected in these single working memory tasks was then used in the dual tasks described in the next section. The two tasks had the same structure but required participants to hold in memory a different type of information (either visuo-spatial or verbal). Participants viewed a first set of elements that had to be held in memory (they were either colored squares or letters), followed by a display showing two arrays of dots (that had to be ignored) and then by a second set of elements, to be compared with the first one. Next, two questions appeared onscreen: the first question “number?” had to be ignored and bypassed by pressing the spacebar, then the second question “same or different?” appeared onscreen and participants were asked to judge whether the second set of elements displayed and the one held in memory were identical or not by pressing the letter “e” or “x” on the keyboard, respectively.

**FIGURE 1:**
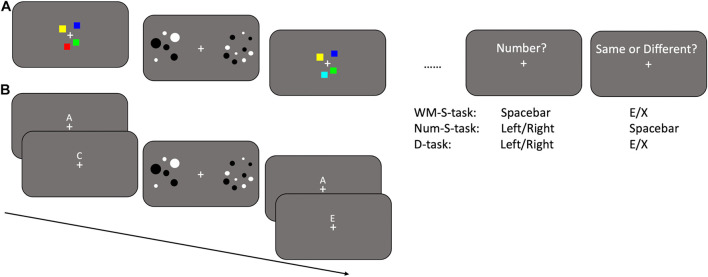
Paradigm and stimuli. Representation of the stimuli used in the visuo-spatial single and dual tasks **(A)** and verbal single and dual tasks **(B)**. Possible responses in the single working memory tasks (WM-S-task), single numerosity (Num-S-task) and dual (D-task) tasks are shown on the right below the relative questions.

In the visuo-spatial working memory task (Figure 1A), the stimuli consisted in arrays of either two or four squares (0.4° side) of different colors (selected randomly between red, green, blue, yellow, and cyan). The number of squares presented (either two or four) was varied to modulate the working memory load (either low or high load, respectively). The arrays of squares were presented in the center of the screen for 500 ms. On every trial the spatial location of the squares was randomly selected within a virtual circle of 3° diameter, not overlapping with the fixation point. In half of the trials the second array of squares was identical to the first one, while in the other half the color of one square was changed. In the verbal working memory task (Figure 1B), participants were presented with sequences of either two or four letters (corresponding to low or high load, respectively). The letters were randomly selected between A, B, C, D, E, and F and were presented just above the fixation point. Each letter stayed onscreen for 500 ms and was immediately replaced by the following one. In half of the trials the second sequence of letters was identical to the first one, while in the other half one letter was replaced by another one not yet presented. Participants performed 96 trials for each task, after which data were analyzed to check that performance was approximately comparable across tasks for both the low and high load conditions (corresponding to the comparison of two or four elements). If the proportion of correct responses in the verbal working memory task largely differed from the one obtained in the visuo-spatial working memory task, then the number of letters displayed was decreased or increased by one element and the participant was tested again with the verbal working memory task. The difference between the two tasks was minimized by selecting, for each participant, the number of letters that allowed equating accuracy across the two tasks. This resulted in selecting 2 letters for the low load condition (except for two participants for which 3 letters were selected) and 4 letters for the high load condition (except for one participant for which 3 letters were selected and three participants for which 5 letters were selected). With this selection the proportion of correct responses between the two tasks did not differ by more than 0.1 (in both directions).

### Single Numerosity and Dual Tasks

After the baseline numerosity and single working memory tasks, participants performed two single numerosity and two dual task experiments in randomized order (Figures 1A,B). The structure of the tasks was the same as for the previously described single working memory tasks: participants were presented with the first set of elements (either squares or letters), then with two arrays of dots and finally with the second set of elements. Following the presentation of these stimuli, the two questions “number?” and “same or different?” appeared onscreen. In the two single tasks, participants had to ignore the set of stimuli (either squares or letters) presented both before and after the arrays of dots and respond to the question “number?” by pressing the left or right arrow to indicate which array contained more dots. The question “same or different?” had then to be ignored by pressing the spacebar. These two single numerosity tasks differed from the baseline numerosity task by the presence of visual stimuli (squares and letters) presented before and after the arrays of dots.

In the two dual tasks, participants were instructed to perform the primary task, that is determining which array of dots was more numerous and then to perform the secondary task in which they had to say whether the first and the second sets of stimuli (either squares or letters) presented before and after the arrays of dots were identical or not. The response to the primary task had to be provided after the question “number?” by pressing the left or right arrow, while the response to the secondary task had to be provided after the question “same or different?” by pressing the letter “e” or “x” on the keyboard.

For each task (two single and two double tasks), participants performed three sessions, with the same number of trials as the ones detailed in the baseline numerosity discrimination task. Half of the trials tested the low load condition, and the other half the high load condition in the secondary task.

### Arithmetic Test

Participants were also tested with an arithmetic test taken from the Italian battery for developmental dyscalculia (Biancardi and Nicoletti, 2004). Participants were asked to respond as quickly and as accurately as possible to a set of single-digit arithmetical operations. The operations were orally presented by the experimenter, who also started the time recording with a chronometer as soon as the question was completely formulated. Time recording was stopped when participants spell out the result. The single-digit operations included 16 multiplications, 6 additions and 6 subtractions. Reaction time (RT) and response accuracy were used to calculate the inverse efficiency score (IES) as the mean RT divided by the proportion of correct responses.

### Analysis

For the working memory tasks, we calculated the proportion of correct responses after splitting the data for the two load conditions (low and high load) and we compared them by means of repeated measures ANOVAs and *t*-tests.

To quantify the precision of numerosity judgments, we plotted the percentage of test trials with “greater than reference” responses against the log-transformed difference between test and reference and fitted it with a cumulative Gaussian function using Psignifit toolbox (Schütt et al., 2016) available at https://github.com/wichmann-lab/psignifit. The point of subjective equality (PSE) was estimated at the 50% point, while the just noticeable difference (JND) was estimated as the difference between the 50 and 75% points.

Next, we estimated the perceptual bias to quantify the influence of the unattended dimension (average size) on the numerosity judgments. To this aim, we fitted participants’ responses after splitting the dataset for the different magnitudes (small or big) of the unattended size dimension. This means that the “unattended small” and the “unattended big” trials had a small (0.25°) or big (0.5°) average item diameter, respectively. A shift of the psychometric curve away from 0 would indicate a bias from the unattended dimension, meaning that the arrays’ average item size induced over- or underestimation of numerosity. For each participant, we fitted the data after splitting for the magnitude of the unattended dimension and calculated the difference (small-big) between the two PSE estimates (signed bias).

Previous studies found diverging results regarding the direction of the numerosity bias induced by item size, sometimes reporting overestimation (Hurewitz et al., 2006; Nys and Content, 2012) and sometimes underestimation (Ginsburg and Nicholls, 1988; Tokita and Ishiguchi, 2010; Gebuis and Reynvoet, 2012b) of number with big item size. Moreover, even within the same study, the direction of the bias is not always the same in all participants (DeWind et al., 2015; Castaldi et al., 2018). Following Castaldi et al. (2018), we additionally calculated the unsigned bias as a measure of the degree of the interference (irrespective of its direction) of the unattended dimension on the numerosity judgments, by taking the absolute value of the signed bias.

The precision of numerosity judgments and biases measured in the different tasks were compared by means of repeated measures ANOVAs and *post hoc* tests. One sample *t*-tests were used to evaluate whether signed biases were significantly different from 0. However, strong but opposite sign effects at the individual participant level could cancel each other out, leading to absence of average bias. We tested whether this was the case by performing individual participant analysis on signed biases. Psignifit toolbox allows to compute Bayesian confidence intervals (credible intervals) based on the posterior marginal densities of the psychometric curve’s parameters. From individual participants’ posterior distributions for unattended small and big’s PSEs, we obtained the 95% credible interval of the difference and the probability p corresponding to 1- the confidence level for which the credible interval would include 0. If *p* < 0.05, 0 was outside the 95% credible interval and the given participant’s bias was considered reliably different from 0.

Finally, on the data collected in the current experiment, we performed correlation analyses based on Pearson correlation to evaluate the relation between the bias and the participants’ arithmetical performance defined as the IES measured with the arithmetic test.

To evaluate the reliability of the current results, we additionally performed Bayesian statistical analysis using JASP (JASP Team, 2020). Hypotheses were tested two-sided using a default prior distribution. For Bayesian ANOVA, models were ordered by their predictive performance relative to the best model. Inclusion Bayes factors resulting from the analysis of effects across “all matched models” are reported for main effects and interaction terms (van den Bergh et al., 2019). Bayes factors are reported in logarithmic base 10 units (LogBF) and their absolute values should be interpreted as providing anecdotal (0–0.5), substantial (0.5–1), strong (1–1.5), or very strong (>1.5) evidence, in favor of the alternative hypothesis if positive, or the null hypothesis if negative.

## Results

### Comparison Between Baseline and Single Numerosity Tasks

In the baseline and in the two single tasks, participants performed a numerosity discrimination task, while ignoring other visual stimuli that were presented. We compared Weber fractions and biases induced by the unattended size dimension measured during numerosity discrimination and found that these variables did not differ across conditions, suggesting that the mere presence of visual stimuli in the two single tasks had no impact on numerosity judgments and these tasks could thus be considered as baseline conditions (see Supplementary Analyses).

### Comparison Between Single Numerosity and Dual Tasks

In the two dual tasks, participants viewed the same images that were shown in the single tasks and were instructed to perform both a numerosity discrimination task (primary task) and a working memory task (secondary task). In the working memory task, participants were asked to judge whether two sets of items presented before and after the dot arrays were the same or not. Items were either a set of squares (visuo-spatial working memory task) or a sequence of letters (verbal working memory task). The working memory tasks had two difficulty levels, requiring participants to hold in memory fewer or more items (low vs. high load conditions).

#### Precision of Numerosity Judgments

**FIGURE 2:**
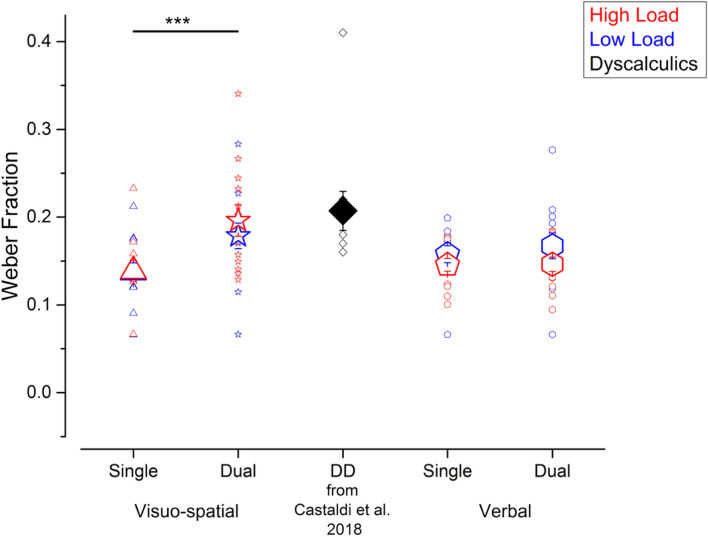
Participants’ precision in the numerosity discrimination tasks. Wfs measured in the different experiments performed in the current study (blue and red symbols) and for the DD participants tested in Castaldi et al. (2018) (black symbols, for qualitative comparison). Blue and red symbols identify the Wfs measured when fewer or more elements were presented before and after the arrays of dots (corresponding to the low and high load conditions in the working memory task). Large and small symbols indicate the average ± SEM and individual Wfs, respectively. ***p < 0.005.

We evaluated participants’ precision in the numerosity discrimination task, as indexed by the Weber fraction (Wf). Performing a secondary task increased participants’ Wfs, irrespective of the working memory load, and most strongly when participants performed the visuo-spatial compared to verbal working memory task (Figure 2). Specifically, Wfs measured in the visuo-spatial dual task were on average larger (low load: 0.18 ± 0.05; high load: 0.19 ± 0.06), compared to the ones measured during the verbal dual task (low load: 0.17 ± 0.05; high load: 0.15 ± 0.03), and both single tasks (visuo-spatial low load: 0.14 ± 0.04; visuo-spatial high load: 0.14 ± 0.04; verbal low load: 0.16 ± 0.03; verbal high load: 0.15 ± 0.03). We entered Wfs in a three-way repeated measures ANOVA with condition (2 levels: single vs. dual task), working memory type (2 levels: visuo-spatial vs. verbal working memory) and load (low vs. high load) as factors. There was a significant interaction between condition and working memory type [*F*(1, 11) = 5.2; *p* = 0.04, LogBF = 0.8], while the triple interaction between condition, working memory type and load [*F*(1, 11) = 1.04; *p* = 0.33, LogBF = –0.3], as well as the interaction between load and the other two factors [condition × load: *F*(1, 11) = 0.09; *p* = 0.76, LogBF = –0.5; working memory type × load: *F*(1, 11) = 1.6; *p* = 0.23, LogBF = –0.1] were not significant. *Post hoc* tests showed that Wfs measured in the visuo-spatial dual task were significantly larger with respect to those measured in the visuo-spatial single task [*t*(11) = –3.9, *p* = 0.004, LogBF = 0.8]. On the contrary, Wfs measured in the verbal dual task were not significantly larger with respect to the verbal single task [*t*(11) = –0.4, *p* > 0.99, LogBF = –0.4]. The Wf differences between the two single and the two dual tasks, respectively, were not significant [single tasks: *t*(11) = 0.99, *p* > 0.99, LogBF = –0.3; dual tasks: *t*(11) = –2.2, *p* = 0.23, LogBF = 0.3].

Overall, compared to the single tasks, participants’ precision when estimating numerosity was affected by the concurrent dual task, especially when visuo-spatial and not verbal stimuli had to be held in memory. On average, numerosity precision in the visuo-spatial dual task approached the one measured in a group of adults with DD measured in a previous study (Castaldi et al., 2018).

#### Interference From the Unattended Size Dimension

**FIGURE 3:**
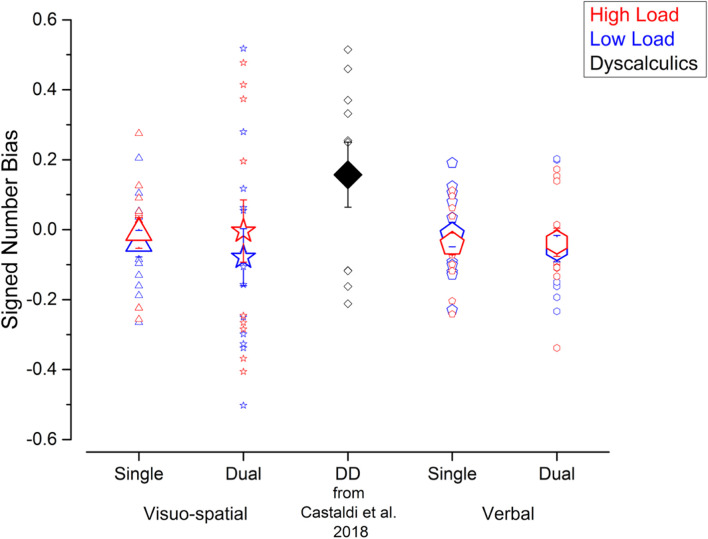
Interference from the unattended dimension in the numerosity discrimination tasks – signed bias. Signed biases measured in the different experiments performed in the current study (blue and red symbols) and in a group of DD participants (black symbol, for qualitative comparison) tested by Castaldi et al. (2018). Symbol size and color correspond to the average and individual biases and to the different loads in the secondary task.

Next, to test whether the unattended size dimension interfered with participants’ judgments, we evaluated the biases defined as the signed difference of the PSEs for psychometric curves fitted using trials with small or big average item size. Figure 3 shows that average signed biases did not seem to be affected by condition and were close to zero for both single (visuo-spatial low load: –0.04 ± 0.14; visuo-spatial high load: –0.009 ± 0.16; verbal low load: –0.02 ± 0.13; verbal high load: –0.04 ± 0.11) and dual (visuo-spatial low load: –0.08 ± 0.3; visuo-spatial high load: –0.004 ± 0.32; verbal low load: –0.05 ± 0.14; verbal high load: –0.04 ± 0.15) tasks. A three-way repeated measures ANOVA on signed biases with condition, working memory type and load as factors showed a significant interaction between working memory type and load [*F*(1, 11) = 5.3; *p* = 0.04, LogBF = –0.4], but *post hoc* tests were all not significant. Other interactions and main effects were not significant [condition: *F*(1, 11) = 0.18; *p* = 0.68, LogBF = –0.7; working memory type: *F*(1, 11) = 0.005, LogBF = –0.7; *p* = 0.95; load: *F*(1, 11) = 2.1; *p* = 0.17, LogBF = –0.5; condition × working memory type: *F*(1, 11) = 9^∗^10^–5^; *p* = 0.99, LogBF = –0.5; condition × load: *F*(1, 11) = 1.09; *p* = 0.32, LogBF = –0.4; condition x working memory type × load: *F*(1, 11) = 3^∗^10^–5^; *p* = 0.99, LogBF = –0.5]. One sample *t*-tests against zero were not significant (all ps > 0.1), consistent with biases measured in all conditions being close to zero on average across participants. However, the absence of a group average bias can be potentially due to strong but opposite effects at the single participant level which cancel each other out. Analysis at the individual participant level (using Bayesian statistics, see methods for details) showed that this was indeed the case for the visuo-spatial dual task: in this condition the signed bias was reliably different from 0 in six and eight participants for the low and high load trials, respectively. Within the participants who showed a bias reliably different from 0, four out of six participants and five out of eight participants for the low and high load conditions, respectively, tended to overestimate numerosity when the unattended size dimension was small and to underestimate numerosity when the unattended size dimension was big, while the remaining participants showed the opposite effect. In the other conditions, the signed biases were reliably different from 0 only in very few participants (3 participants for both loads of the visuo-spatial single task, 1 participant for both loads of the verbal single task and 2 and 1 participants for the low and high load conditions of the verbal dual task). Overall, these results suggest that although the average signed bias is close to 0 for all conditions, reliable effects of either positive or negative direction were observed in individual participants in the case of the visuo-spatial dual task.

These results shown by the current participants without DD in the visuo-spatial dual task condition resemble the ones previously obtained in a group of DD participants (Castaldi et al., 2018): the average signed bias in the DD group was not significantly different from zero, but analysis at the individual participant level showed that the signed biases were reliably different from 0 in all DD participants.

**FIGURE 4:**
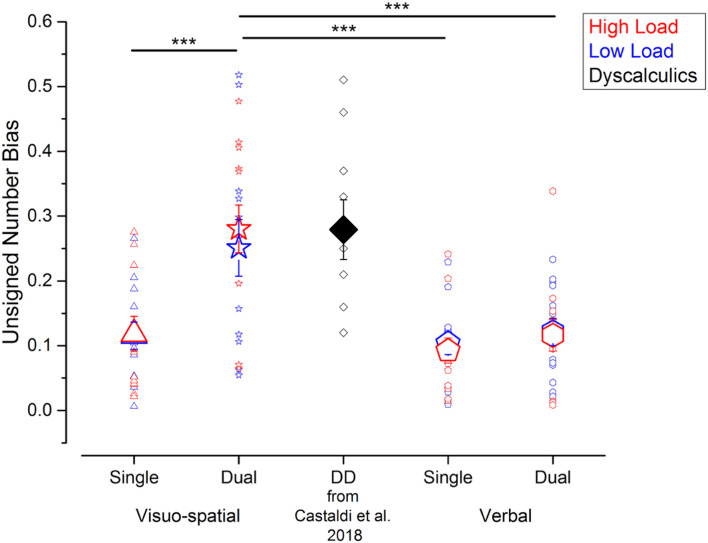
Interference from the unattended dimension in the numerosity discrimination tasks – unsigned bias. Unsigned biases measured in the different experiments performed in the current study (blue and red symbols) and in a group of DD participants (black symbol, for qualitative comparison) tested by Castaldi et al. (2018). Symbol size and color correspond to the average and individual biases and to the different loads in the secondary task. ***p < 0.005.

To compare the strength of the biases irrespective of their direction, we next evaluated the unsigned biases defined as the absolute difference of the PSEs for psychometric curves fitted using trials with small or big average item size. Unsigned biases were affected by the secondary task, in a similar way as Wfs (Figure 4). Unsigned biases measured in the visuo-spatial dual task were on average higher (low load: 0.25 ± 0.16; high load: 0.28 ± 0.13) than the ones measured during the verbal dual task (low load: 0.12 ± 0.07; high load: 0.12 ± 0.09) and both the single task conditions (visuo-spatial low load: 0.11 ± 0.07; visuo-spatial high load: 0.12 ± 0.09; verbal low load: 0.10 ± 0.06; verbal high load: 0.09 ± 0.07). The three-way repeated measures ANOVA on unsigned biases with condition, working memory type and load as factors revealed a significant interaction between condition and working memory type [*F*(1, 11) = 39.5; *p* < 0.001, LogBF = 1.4], while the triple interaction between condition, working memory type and load [*F*(1, 11) = 0.07; *p* = 0.79, LogBF = –0.5], and the interaction between load and the other two factors [condition × load: *F*(1, 11) = 0.21; *p* = 0.66, LogBF = –0.5; working memory type x load: *F*(1, 11) = 0.5; *p* = 0.49, LogBF = –0.5] were not significant. *Post hoc* tests showed that the unattended size dimension biased participants’ judgments significantly more during the visuo-spatial dual task than the visuo-spatial single task [*t*(11) = –6.8, *p* < 0.001, LogBF = 1.1]. On the contrary, unsigned biases were not significantly stronger when participants were involved in the verbal dual task with respect to the verbal single task [*t*(11) = –0.9, *p* > 0.99, LogBF = –0.5]. Unsigned biases were also significantly stronger when participants performed the visuo-spatial dual task compared to the verbal single [*t*(11) = 4.6, *p* = 0.001, LogBF = 0.7] and verbal dual tasks [*t*(11) = 4.5, *p* = 0.003, LogBF = 0.9]. Unsigned biases measured in the two single tasks did not differ from each other [*t*(11) = –0.6, *p* > 0.99, LogBF = –0.5).

Overall, compared to the other conditions measured here, the unattended size dimension biased participants’ judgment most strongly during the visuo-spatial dual task, in which case the degree of bias approached the one previously observed in a group of adults with DD in a single task (Castaldi et al., 2018).

### Comparison Between Single and Dual Working Memory Tasks

**FIGURE 5:**
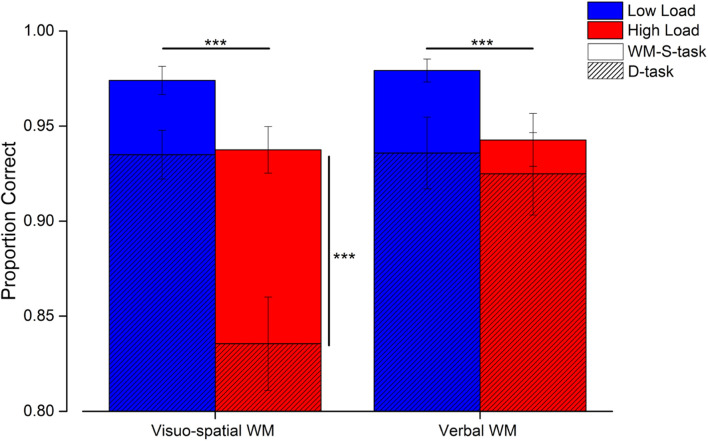
Results of the working memory tasks. Average performance in the working memory tasks in the single (solid bars) and dual tasks (hatched bars) for both the low (blue) and high (red) load trials. Error bars are SEM. ***p < 0.005.

Participants performed two single working memory tasks (WM-S-task) to select the number of elements subsequently used that matched the verbal and visuo-spatial working memory load (Figure 5 light and dark gray bars). In both of these single working memory tasks, the proportion of correct responses was overall very high and comparable (visuo-spatial working memory task low load: 0.97 ± 0.03, high load: 0.94 ± 0.04; verbal working memory task low load: 0.98 ± 0.02, high load: 0.94 ± 0.05), confirming that task difficulty was successfully matched across the two systems (see Supplementary Material). Working memory performance in the dual task conditions was still relatively high (Figure 5, hatched bars), yet lower than that measured in the single working memory tasks (visuo-spatial working memory task low load: 0.94 ± 0.05, high load: 0.84 ± 0.09; verbal working memory task low load: 0.94 ± 0.07, high load: 0.92 ± 0.08).

The proportion of correct responses made in the working memory tasks was entered in a three-way repeated measures ANOVA with condition (2 levels: single and dual task), working memory type (2 levels: visuo-spatial and verbal working memory task) and load (2 levels: low and high) as factors. The triple interaction between working memory type, load and condition was significant [*F*(1, 11) = 21.7; *p* < 0.001, LogBF = 0.8]. Subsequent *post hoc* tests showed that the proportion of correct responses decreased during the high (but not low) load trials of the visuo-spatial dual task with respect to the corresponding single task trials [high load single vs. dual task: *t*(11) = 6.2, *p* < 0.001, LogBF = 2.2; low load single vs. dual task: *t*(11) = 2.4, *p* = 0.69, LogBF = 1.1; Figure 5, vertical significant bar]. On the other hand, performing the verbal dual task did not significantly decrease the proportion of correct responses with respect to the verbal working memory single task, neither for the low nor for the high load trials [low load single vs. dual task: *t*(11) = 2.6, *p* = 0.38, LogBF = 0.3; high load single vs. dual task: *t*(11) = 1.08, *p* > 0.99, LogBF = –0.3]. In the dual tasks, the proportion of correct responses in the high load condition of the visuo-spatial working memory task was significantly lower with respect to the high load condition of the verbal working memory task [*t*(11) = 6.2, *p* < 0.001, LogBF = 1.6]. This difference was not significant for the low load trials [*t*(11) = 0.06, *p* > 0.99, LogBF = –0.5].

In sum, these results suggest that performing a concurrent numerosity task interferes with performance in a visuo-spatial working memory task, especially when load levels are relatively high, but this was not the case with a verbal working memory task.

### Correlation Analyses

**FIGURE 6:**
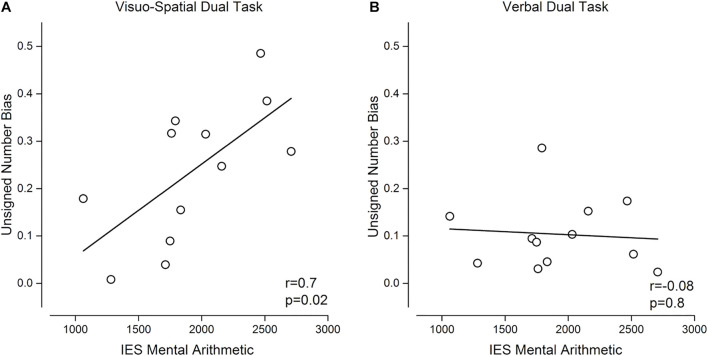
Relation between unsigned bias and mental arithmetic skills. Pearson correlation between unsigned bias when discriminating numerosity during the visuo-spatial **(A)** or verbal **(B)** dual tasks and inverse efficiency score (IES, see section “Arithmetic Test”) in mental arithmetic.

Finally, we performed exploratory correlation analyses to test whether the tendency to show enhanced interference from non-numerical dimensions under concurrent working memory load was related to arithmetical abilities (Figure 6). We observed a significant positive correlation between the size of unsigned biases during the numerosity discrimination task and IE score for calculation, indicating that participants with better arithmetical abilities were those whose numerosity judgments were less biased by the unattended dimension (*r* = 0.7, *p* = 0.02, LogBF = 0.6, Figure 6A). Interestingly, arithmetic abilities were predicted by the size of unsigned biases only when numerosity discrimination was performed during the visuo-spatial, and not during the verbal, dual task: the correlation between unsigned bias during the verbal dual task and IE score for calculation was not significant (*r* = –0.08, *p* = 0.8, LogBF = –0.5, Figure 6B).

## Discussion

In the current study we investigated whether resources required for visuo-spatial working memory that explicitly encode the locations of multiple items in the visual scene might also be crucial to precisely and veridically perceive larger numerosities without bias from non-numerical quantities. We therefore measured not only numerosity precision, but also perceptual biases (interference) during a dual task design, and directly tested which specific type of working memory (visuo-spatial vs. verbal) showed bidirectional interference with numerosity processing. We further explored whether these shared resources might also be relevant for arithmetical abilities.

In line with our hypothesis of a shared resource, we found that participants’ threshold and the interference from the unattended dimension during a numerosity discrimination task increased when participants performed a concurrent visuo-spatial (but not verbal) working memory task. This interference was bidirectional: performing a numerosity discrimination task also degraded performance during the visuo-spatial working memory task. Finally, we found that the degree of interference from the unattended dimension on numerosity judgments during the concurrent visuo-spatial (but not verbal) task predicted interindividual differences in arithmetical skills.

Our results fit well with a recent study that also suggested a role of visuo-spatial working memory capacity in the extent to which participants’ numerosity judgments rely on non-numerical dimensions (Lee and Cho, 2019). In that study, participants were assigned to low or high working memory groups based on their memory span (measured with dedicated tasks) and asked to numerically compare arrays of dots (12–40 dots) in which the non-numerical dimensions varied either congruently or incongruently with numerosity. Numerosity judgments of participants included in the low visuo-spatial working memory group were more influenced by non-numerical dimensions (size, total surface area and density) compared to those of participants in the high visuo-spatial working memory group, while the same was not observed when splitting the groups based on their verbal working memory span. While this plausibly suggested that the susceptibility to non-numerical interference during numerosity judgments depends on the capacity of the participants’ visuo-spatial but not verbal working memory, uncontrolled domain general abilities other than working memory may have accidentally characterized the two subgroups tested in that study. Manipulating the engagement of visuo-spatial and verbal working memory resources in the same participants, as done in the current study, is therefore necessary to establish their relation with numerosity processing more unambiguously.

The present results also extend evidence from previous studies suggesting that a mechanism of visual indexing of multiple objects supports both visuo-spatial working memory and enumeration (Melcher and Piazza, 2011; Piazza et al., 2011; Knops et al., 2014) by showing that this mechanism might also operate at higher numerosities. The supposed underlying mechanisms of a saliency map has been simulated in computational studies using networks consisting of interconnected nodes which exhibit recurrent self-excitation and lateral inhibition (Roggeman et al., 2010; Knops et al., 2014; Sengupta et al., 2014). Each node corresponds to a neural population encoding an object location or feature and interacting with the other nodes through lateral inhibition. High levels of lateral inhibition lead to low noise levels and precise representations, but also to a small capacity of the map. On the contrary, lower levels of lateral inhibition lead to higher noise levels, coarser representations, and higher capacity limits. Thus, capacity limits are not fixed, but can vary depending on the representational precision required by the task, which can top-down modulate the level of lateral inhibition in the saliency map (Roggeman et al., 2010; Melcher and Piazza, 2011; Sengupta et al., 2014). The predictions of these models have found support in neurophysiological and fMRI studies (Bisley, 2003; Roggeman et al., 2010; Knops et al., 2014). The lateral intraparietal cortex (LIP) of macaque monkeys was found to represent the attended locations in the visual fields (Bisley, 2003) and its homolog region in humans showed signatures of saliency map models, e.g., by modulating voxels’ response profiles depending on the representational precision required by the specific task at hand (Roggeman et al., 2010; Knops et al., 2014). Knops et al. (2014) showed participants a variable number of oriented Gabor gratings and asked them to either remember and compare their orientation or to enumerate them. They found that the average response profiles and the pattern of activation of the same set of voxels in the posterior parietal cortex (PPC) changed as a function of the task. While Knops et al. (2014) mainly investigated small numerosities, other studies have suggested that the capacity of the saliency maps need not be limited to few items within the subitizing range but can extend even to higher numerosities depending on task requirements and the level of top-down attention directed to individual items. Roggeman et al. (2010) found that when participant were asked to perform a numerosity estimation task on numbers above the subitizing range, the activity in IPS regions increased up to eight items and then slightly decreased (as for salience map models with medium inhibition settings), whereas when participants were performing a less demanding pattern detection task, the activity in IPS regions showed a V-shape, decreasing from four to eight or sixteen items and then increasing again (up to 64), as also found for a model with low inhibition settings. Finally, Sengupta et al. (2014) confirmed that changing the level of inhibition between nodes allowed the same network architecture to account for number discrimination in both the subitizing and estimation ranges. Altogether, these studies suggest that the PPC may host a mechanism which might be conceived as a saliency map contributing to both enumeration and visual working memory. This mechanism might allow to form a representation of the locations of a number of items, which would be coarser/less precise for larger numbers, but sufficient for extraction of approximate numerosity. In relation with the findings of the current study, we suggest that this mechanism led participants to localize and segregate items quite accurately during the baseline and single tasks. However, during the dual task this system might have been saturated by the need to precisely represent the stimuli of the visuo-spatial working memory task, leading participants’ numerosity judgments to rely more on some kind of coarser, undifferentiated summary statistics representation of the visual arrays. For example, reliance on total energy or surface area might have led participants to overestimate numerosities with big dot sizes. On the contrary, reliance on the relative amount of energy in high and low spatial frequencies of the image, as predicted by a model linking numerosity to texture density processing (Dakin et al., 2011; Tibber et al., 2012), might have led them to overestimate numerosities with small dot sizes.

If the extraction of veridical numerosity estimates relies on indexing spatial locations of objects by a mechanism potentially involving recurrent processing, then it can be predicted that displaying stimuli for a longer time on screen should allow participants to discriminate numerosities more accurately and to provide less biased numerosity judgments. This has partially been observed: Inglis and Gilmore (2013) found that numerosity precision increased with the exposure to the stimulus display, and that this effect could not be explained by differences in the onset to decision latencies (and presumably not even by the adoption of counting strategies given that the effect was observed also for latencies below 1 s). Future studies should test whether longer presentation time also reduces interference from non-numerical dimensions on numerosity judgments. Further work may also manipulate the saliency of individual items and test in how far estimates of larger numerosities in such situations are indeed well explained by saliency map models or would require still somewhat different mechanisms.

The results of the current study clearly indicate that it is not any kind of working memory load irrespective of domain, but more specifically the visuo-spatial component which shares resources with numerosity judgments. The importance of visuo-spatial rather than verbal or auditory resources for a precise numerosity representation has been observed also during other tasks and for other cognitive functions than memory. For example, in a number line task, in which participants had to spatially map the relative location of arrays of different numerosities onto a line defining a numerical interval, participants’ responses changed from being linearly distributed to logarithmic-like if they had to perform a concomitant visuo-spatial, but not auditory, task (Anobile et al., 2012a,b). Depriving visuo-spatial attention by means of attentional blink or dual tasks paradigms also affected the precision of numerosity estimation (Vetter et al., 2008; Burr et al., 2010; Anobile et al., 2012b; Pomè et al., 2019), while this was much less observed when the distractor task required directing attention to auditory stimuli (Pomè et al., 2019). Interestingly, although the strongest detrimental effects of attentional deprivation on numerosity estimation have been reported for low numerosities in the subitizing range, some smaller but consistent effects have been observed also for higher numerosities: Depriving visuo-spatial attention increased the degree of underestimation (Vetter et al., 2008; Burr et al., 2010; Anobile et al., 2012b) and decreased numerosity estimation precision also for numerosities beyond the subitizing range (Vetter et al., 2008; Pomè et al., 2019). Splitting visuo-spatial attention during numerosity adaptation by simultaneously presenting a numerically neutral adapter alongside with the real one led to underestimation of the real adaptor and to a consequent reduction of the adaptation effect (Grasso et al., 2021b,a).

The fact that deprivation of both visuospatial attention and working memory resources can affect aspects of numerosity perception fits well with a supposed functional overlap in the mechanisms of spatial working memory and spatial selective attention which may both be based on the same spatial saliency map (Awh and Jonides, 2001; Deco and Rolls, 2005). The current results extend the existing literature showing that engaging visuo-spatial working memory resources does not merely make numerosity estimates more noisy overall which could have been one possibility, but also increases the perceptual biases.

The fact that loading visuo-spatial working memory in neurotypical participants qualitatively simulated previous findings obtained in DD adults compared to controls and the finding that in the current study the bias correlated with interindividual differences in arithmetic abilities, make us speculate that the common resource that supports both visuo-spatial working memory and numerosity extraction may also play an important role in arithmetical learning, and be potentially impaired in DD. The limited visuo-spatial working memory capacity, the lower precision, and the enhanced reliance on non-numerical dimensions during numerosity discrimination tasks often observed in DD individuals, which are often separately emphasized by alternative and competing explanatory accounts of this disorder, could thus be interdependent phenomena and reflect a weakness of the same system. The fact that this system specifically supports visuo-spatial but not verbal working memory, is in line with the previously reported correlation between numerosity impairments and visuo-spatial, but not verbal, working memory performance in DD children (Bugden and Ansari, 2016). It is also in line with the selective impairment of visuo-spatial working memory characterizing the “pure” DD subtype (i.e., without associated reading problems, Szûcs, 2016).

The parietal regions exhibiting properties of a saliency map (Knops et al., 2014) are also modulated by attention to (high) numerosities as opposed to other non-numerical dimensions (Castaldi et al., 2019) and the pattern of activity read out from these regions correlates with numerosity precision (Lasne et al., 2018). Areas which are likely overlapping or nearby are recruited during visuo-spatial working memory and arithmetic tasks (Zago et al., 2008; Castaldi et al., 2020c; Matejko and Ansari, 2021) and present functional abnormalities in DD individuals during both magnitude discrimination and visuo-spatial working memory tasks (Price et al., 2007; Rotzer et al., 2009). It is thus in theory possible that the neural substrate of the common resource supporting visual working memory and numerosity extraction in parietal cortex is impaired in DD. Nevertheless, the fact that the present study in neurotypical adults yielded qualitatively similar findings to those previously observed in dyscalculics is not necessarily evidence for a shared cause. Future behavioral and imaging studies in dyscalculia may further test this possibility.

## Conclusion

In conclusion, the current study shows that estimating large numerosities veridically relies on resources that are also fundamental for visuo-spatial but not verbal working memory, which may relate to explicitly encoding the locations of multiple objects in the visual scene. Loading visuo-spatial working memory may saturate this system and lead participants’ numerosity estimates to rely more on a coarse, gist-like representation of the visual input which is susceptible to the influence of non-numerical dimensions. Although speculative, it is possible to hypothesize that the difficulties experienced by DD individuals with both numerosity perception and working memory may result from the impairment of the same resources which would explain why low numerosity discrimination precision, enhanced reliance on non-numerical dimensions during numerosity judgments and impaired visuo-spatial working memory often co-occur.

## Data Availability Statement

The raw data supporting the conclusions of this article will be made available by the authors, without undue reservation.

## Ethics Statement

The studies involving human participants were reviewed and approved by the Research Ethics Committee of University Paris-Saclay. The patients/participants provided their written informed consent to participate in this study.

## Author Contributions

EC collected and analyzed the data. All authors contributed to the study concept, experimental design, interpretation of results, manuscript preparation, and approved the final version of the manuscript.

## Conflict of Interest

The authors declare that the research was conducted in the absence of any commercial or financial relationships that could be construed as a potential conflict of interest.

## Publisher’s Note

All claims expressed in this article are solely those of the authors and do not necessarily represent those of their affiliated organizations, or those of the publisher, the editors and the reviewers. Any product that may be evaluated in this article, or claim that may be made by its manufacturer, is not guaranteed or endorsed by the publisher.
