# Supplementary material for: Resources Underlying Visuo-Spatial Working Memory Enable Veridical Large Numerosity Perception
Source: Front Hum Neurosci. 2021 Nov 15;15:751098. doi: 10.3389/fnhum.2021.751098 (PMC8634845; doi:10.3389/fnhum.2021.751098)
Supplement: Supplementary file 1 [file Data_Sheet_1.docx]

**Comparison between baseline and single task numerosity tasks**

In the baseline and in the two single task experiments participants performed a numerosity discrimination task, while in the single tasks in addition ignoring other visual stimuli that were presented.

We evaluated participants’ precision in the numerosity discrimination task, as indexed by the Weber fraction (Wf) and found that it was very similar across all conditions, being equal to 0.16±0.02 for the baseline, 0.14±0.04 for the visuo-spatial single task and 0.15±0.02 for the verbal single task. To compare the Wfs measured in these three conditions we entered them into a one-way ANOVA with condition as factor. There was no significant main effect of condition (F(2,22)=2.12; p=0.14, LogBF=-0.1), suggesting that the mere presence of visual stimuli in the two single tasks had no impact on participants’ precision during numerosity discrimination.

Next, to test whether the unattended size dimension biased participants’ responses in the baseline and single task experiments, we evaluated the signed and unsigned difference of the PSEs when the psychometric curves were fitted using trials with small or big average item size. Signed biases were close and not significantly different from zero and equal to 0.01±0.12 for the baseline (t(11)=0.37, p=0.7), -0.02±0.12 for the visuo-spatial single task(t(11)=0.72, p=0.5) and -0.03±0.11 for the verbal single task(t(11)=0.9, p=0.4). Theone-way ANOVA revealed no main effect of condition (F(2,22)=0.35; p=0.70, LogBF=-0.5). Analysis at the level of single participants showed that the signed biases in the baseline condition were reliably different from 0 only in three participants, similar to the number of participants in the two single tasks (see main text).Unsigned biases were equal to 0.08±0.08 for the baseline, 0.1±0.05 for the visuo-spatial single task and 0.09±0.06 for the verbal single task and ANOVA revealed no main effect of condition (F(2,22)=0.33; p=0.72, LogBF=-0.5). In sum, both average signed and unsigned biases were not affected by the mere presence of additional visual stimuli during the single tasks.

**Comparison between single working memory tasks**

Participants performed two single working memory tasks to select the number of elements subsequently used that matched the verbal and visuo-spatial working memory load (Figure 5 light and dark gray bars in the main text). To confirm that difficulty was matched across tasks and equally modulated by load levels, the proportion of correct responses measured after calibration of difficulty across tasks was entered in a two-way repeated measure ANOVA withworking memory type (2 levels: visuospatialand verbal working memory task) and load(2 levels: low and high) as factors. The interaction between working memory typeand load (F(1,11)=4.8*10-17; p>0.99, LogBF=-0.4) and the main effect of working memory type(F(1,11)=1.0; p=0.34, LogBF=-0.4) were not significant, suggesting that the number of elements selected for each working memory type and load were charging the visuo-spatial and verbal working memory to the same extent. The significant main effect of load (F(1,11)=14.3; p=0.003, LogBF=3.2) and related post-hoc tests showed that the selected number of elements successfully increased the difficulty of the task for both the visuo-spatial and verbal working memory systems (visuo-spatial low vs high load: t(11)=3.2, p=0.03, LogBF=1.1, verbal low vs high load: t(11)=3.2, p=0.03, LogBF=0.6). Overall, these results suggest that the two working memory tasks were matched for difficulty and the selected number of items sufficiently modulated the working memory load to the same extent across the two systems.
